# Supplementary material for: Monocyte Infiltration and Differentiation in 3D Multicellular Spheroid Cancer Models
Source: Pathogens. 2021 Jul 30;10(8):969. doi: 10.3390/pathogens10080969 (PMC8399809; doi:10.3390/pathogens10080969)
Supplement: Supplementary file 1 [file pathogens-10-00969-s001.zip › pathogens-1310898-supplementary.pdf]

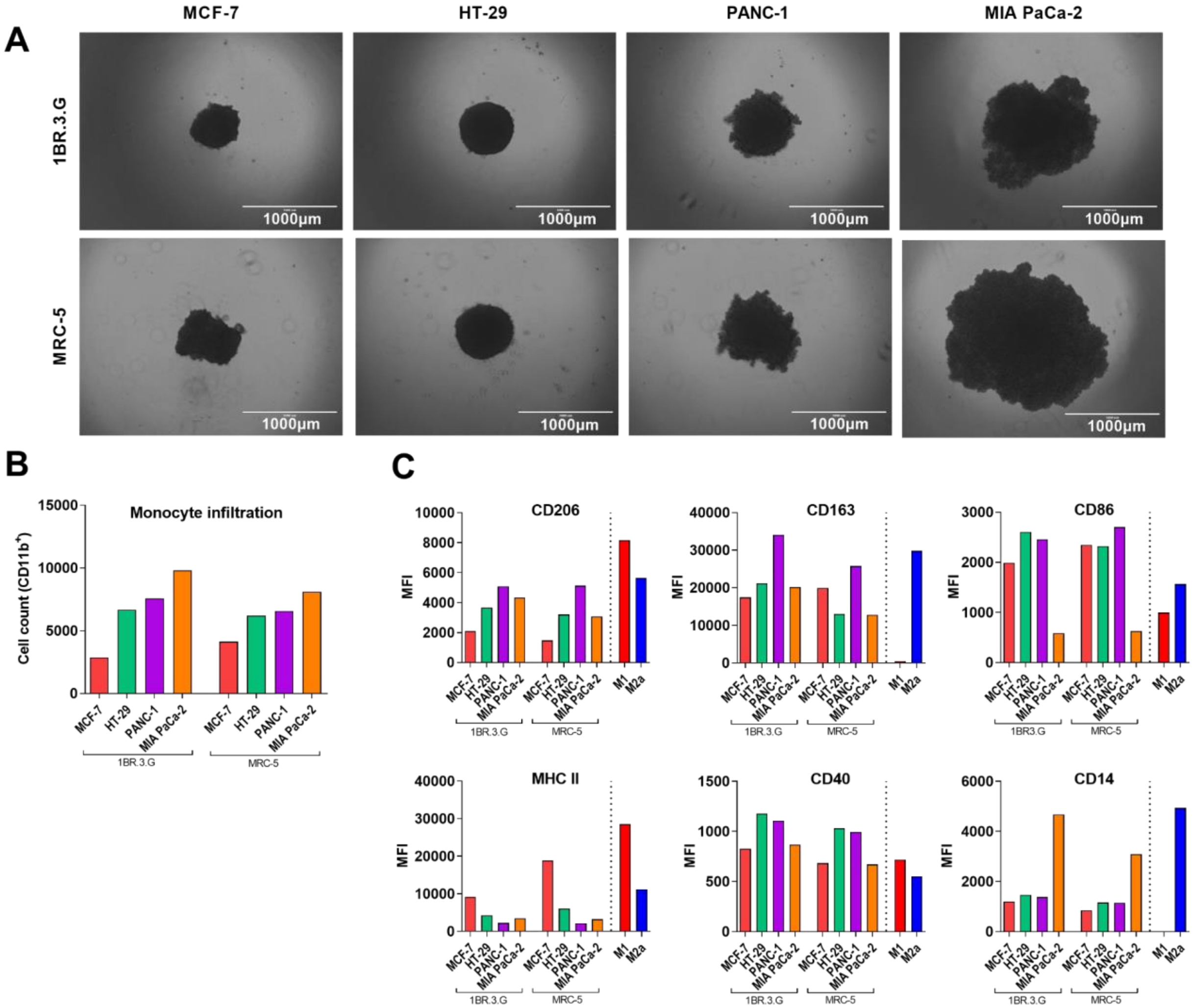

Figure S1. Tumor spheroids co-cultured with 1BR.3.G or MRC-5 cells and donor-derived monocytes. Spheroids were generated from MCF-7, HT-29, PANC-1 and MIA PaCa-2 tumor cells and either 1BR.3.G or MRC-5 fibroblasts. $CD14^+$ monocytes were added to 7 days old spheroids. (a) Bright field images of spheroid structures of 7 days old cancer cell-fibroblast spheroids; (b) Cell surface expression of CD11b in dissociated spheroids measured by flow cytometry; (c) Cell surface expression of macrophage markers MHC II, CD163, CD86, CD206, CD40 and CD14 on live $CD11b^+$ cells by flow cytometry. The spheroid polarized MDMs are compared to control M1 and M2a macrophages. Each bar represents cells from 5 pooled spheroids, except for the M1 and M2a macrophages. Data represent 1 individual experiment with 1 monocyte donor.

Table S1: Profile of macrophages after spheroid-induced polarization over time. Spheroids were generated from MCF-7, HT-29, PANC-1 and MIA PaCa-2 tumor cells and 1BR.3.G fibroblasts. CD14⁺ monocytes were added to 7 days old spheroids and 3, 5 and 7 days later, spheroids were analyzed by flow cytometry. The CD11b⁺ cells were analyzed for surface expression of MHC class II, CD163, CD86, CD206, CD40 and CD14. The CD11b⁺ cells from the spheroid polarized MDMs were compared to control M1 and M2a macrophages generated by cytokine cocktails and unstimulated monocytes. The table display mean MFI values from 5 pooled and dissociated spheroids from each of 4 monocyte donors, except for control M1, M2a macrophages and unstimulated monocytes. MHC class II expression on day 3 only represent data from 1 experiment with 2 monocyte donors.

	Day 3				Day 5				Day 7						
	MCF-7	HT-29	PANC-1	MIA PaCa-2	MCF-7	HT-29	PANC-1	MIA PaCa-2	MCF-7	HT-29	PANC-1	MIA PaCa-2	M1	M2a	Monocytes
CD206	1330	1531	1894	797.4	2529	3296	4137	2322	2849	3087	3981	4319	6171	8041	479.6
CD163	1108	587	797.5	1902	6401	6586	7851	5489	7958	13048	12888	9807	970	24912	18558
CD86	857.8	1027	906.9	497	1589	1826	1712	642	3346	3186	3848	713	1046	1027	857.8
CD40	673.7	491.3	405.3	277.1	1231	857.5	721.4	532	1298	1205	1090	797.7	775.7	748.1	886.3
CD14	777	1010	1113	1884	932.6	1259	1198	2266	893	1224	913.6	2939	315.2	4385	2105
MHC II	1470	1767	1648	2924	4521	2433	1859	1991	6347	2246	1576	1661	24695	4666	23799

Table S2. Profile of macrophages after TCM-induced polarization. 25 % TCM from 2D cultured MCF-7, HT-29 or MIA PaCa-2 with or without cytokines (IL-4 and IL-10) were added to cultures on day 0 or day 6 after seeding of CD14⁺ monocytes. M-CSF is present in the media during the maturation for all TCM-polarized monocytes. 7 days after seeding, cells were harvested and analyzed by flow cytometry. The CD11b⁺ cells were analyzed for surface expression of MHC class II, CD163, CD86, CD206, CD40 and CD14 and compared to control M1 and M2 macrophages generated by cytokine cocktails and unstimulated monocytes. The table display mean MFI values from 4 monocyte donors in total (M1 and M2 macrophages represent 3 donors, and unstimulated monocytes represent 2 donors).

	Day 0 (no cytokines)			Day 6 (no cytokines)			Day 0 (IL4+IL10)			Day 6 (IL4+IL10)					
	MCF-7	HT-29	MIA PaCa-2	MCF-7	HT-29	MIA PaCa-2	MCF-7	HT-29	MIA	MCF-7	HT-29	MIA PaCa-2	M1	M2 (IL-4+IL-10)	Monocytes
CD206	1787	2480	3219	2110	2180	2559	20403	19793	17075	15819	16804	16942	1764	17666	3522
CD163	23332	31335	28867	24762	28371	24571	35372	35899	33809	39299	41205	37582	211.6	48629	5987
CD86	809.1	948.7	832.5	821.3	880	807	320.8	321.6	332.5	410.9	404.9	394.8	3444	461.8	1294
CD40	767.6	801.1	896.2	790.9	787.6	820.8	916.9	884.3	948.2	1046	1020	1104	1469	1058	1967
CD14	4122	5244	7454	4638	4895	5446	2510	2662	2818	5120	5170	5335	51.6	5835	1795
MHC II	19080	15488	8190	17672	17976	15497	13313	12317	10563	4322	4451	3959	42735	4287	42391

Table S3: Cytokine profile of cancer cell-fibroblast spheroids with and without MDMs. Spheroids were generated from MCF-7, HT-29, PANC-1 and MIA PaCa-2 tumor cells and 1BR.3.G fibroblasts. Isolated CD14+ monocytes were added to 7 days old spheroids. 7 days after monocyte addition, supernatant was harvested and a panel of 43 soluble factors was measured using Luminex multiplex Technology. Spheroids without monocytes were included as control and analyzed in the same way. 7 days after seeding of 1×10⁴ monocytes in 96-well plates and subsequent activation, the same cytokines were measured in supernatant from control M1, M2a macrophages generated by cytokine cocktails and unstimulated monocytes. The table display mean pg/ml values from 6 monocyte donors. Data from spheroids without monocytes represent supernatant harvested from 6 individual spheroid cultures.

	MCF-7	MCF-7 + MDMs		HT-29 + MDMs	PANC-1	PANC-1 + MDMs	MIA PaCa-2	MIA PaCa-2 + MDMs	M1	M2a	Monocytes
CCL1	3.683	18.86	12.73	29.09	14.24	29.7	21.24	30.48	543	13.46	18.44
CCL11	3.097	12.37	8.121	17.22	10.02	16.44	15.44	18.94	18.97	22.03	2.965
CCL13	1.727	12.14	7.009	20.77	48.89	80.65	132.3	140.1	25.11	42.74	5.374
CCL15	4.523	23.12	1506	1426	9.878	24.95	16.2	22.09	74.52	12.14	5.185
CCL17	1.222	1.325	0.445	0.733	0.772	0.784	1.955	3.117	5.037	1.257	0.087
CCL19	16.45	110	76.98	176.4	142.8	230.5	288.8	313.5	210.8	51.12	36.24
CCL2	2.171	399.3	1.698	1037	370.6	1009	2.561	470.4	659.5	612.5	169.2
CCL20	0.598	11.77	86.37	120.7	11.08	31.36	2.107	33.83	13.07	0.586	0.440
CCL21	127.7	1127	308.5	1641	1564	1757	521	1620	1163	893	350.3
CCL22	52.36	328	10.33	361.9	7.315	618.4	11.9	504.4	26.65	155.8	1.826
CCL23	0.419	4.23	3.775	9.051	3.006	16.17	7.469	10.47	7.012	3.226	0.500
CCL24	1.689	12159	60.43	19093	25.33	8676	8.704	5235	289.4	219.6	157.4
CCL25	136.7	834.8	415.4	1395	458.1	1580	1790	2899	1428	319.6	168.4
CCL26	25.75	86.44	29.18	136.4	391.9	458.1	86.96	190.8	124.7	53.17	14.94
CCL27	1.226	6.833	4.221	13.53	4.349	10.48	12.59	20.94	18.81	17.19	1.497
CCL3	0.120	49.6	0.725	40.56	0.422	40.89	0.591	6.74	8067	30.11	19.97
CCL7	12.11	224.9	36.02	786.4	110.5	1588	95.18	176.2	149.4	44.37	12.77
CCL8	0.111	251.3	0.185	311.2	1.253	65.67	0.420	5.285	675.5	22.65	5.035
CX3CL1	16.97	35.96	52.13	61.44	1170	964.2	44.25	57.47	29.59	16.51	1.492
CXCL1	75.94	89.89	377.5	431.9	4302	4078	44808	9543	280.8	69.38	26.01
CXCL10	14.08	266.1	400.8	958.2	12.48	118.1	16.23	65.1	1707	14.19	3.713
CXCL11	3.614	16.41	492.9	549.6	12.62	18.36	3.114	7.323	9.063	0.428	0.174
CXCL12	387.4	767.3	533.7	1152	303.5	764.5	222.2	627.6	261.8	64.13	35.75
CXCL13	0.211	1.873	0.622	3.039	1.213	2.426	1.416	2.226	2.729	0.871	0.249
CXCL16	93.99	142.3	71.01	160.2	53.73	143.3	331.1	335.4	19.57	20.73	14.73
CXCL2	11.06	80.75	23.74	109.9	38.67	159.9	1334	2065	66.1	31	8.593
CXCL5	366.5	1980	1274	3103	3327	6011	81686	84605	3460	1447	561.4

CXCL6	1.526	4.364	11.97	17.36	43.59	54.04	265.7	655.9	7.158	1.964	0.648
CXCL8	151.3	2405	605.3	3413	896.2	4799	12580	10778	4461	225.7	547.3
CXCL9	0.579	9.052	6.556	11.75	5.142	9.833	9.177	21.53	32.97	2.218	0.718
GM-CSF	2.276	6.16	6.036	11.38	3.444	8.754	8.831	11.02	19894	3.739	5.697
VEGF	115.8	79.43	1455	1192	839.7	578.2	1389	1266	15.47	17.09	14.03
IFNY	0.860	47.76	35.36	161.9	42.73	75.02	4.731	40.4	470.1	26.81	9.075
IL-10	1.363	8.394	4.863	12.09	11.78	21.85	27.12	29.44	12.38	2.473	2.3
IL-12	0.107	0.076	0.908	0.3698	0.28	0.152	0.327	0.079	0.569	0	0
IL-16	1.565	31.73	8.202	36.84	10.43	38.84	34.12	48.73	26.81	7.808	4.2
IL-1Ra	1.593	3545	131.7	4110	5.617	5831	4.305	2713	430.8	413.7	55.99
IL-1B	0.362	1.909	1.074	3	1.657	3.534	3.212	4.035	2.911	1.023	0.349
IL-2	0.307	1.991	1.236	2.539	1.861	2.861	3.274	3.731	3.392	1.309	0.286
IL-4	0.440	37.19	3.369	42.31	3.949	41.38	11.37	46.75	8.422	14388	2.362
IL-6	71.31	30.51	38.67	55.87	7.294	27.31	26.5	58.35	669.2	4.47	5.024
MIF	44607	54796	179450	114034	118203	104309	692172	923894	1696	1190	1371
TNF-a	12.23	124.4	44.59	203.6	43.33	174.6	146.6	270.6	1166	67.89	43.04

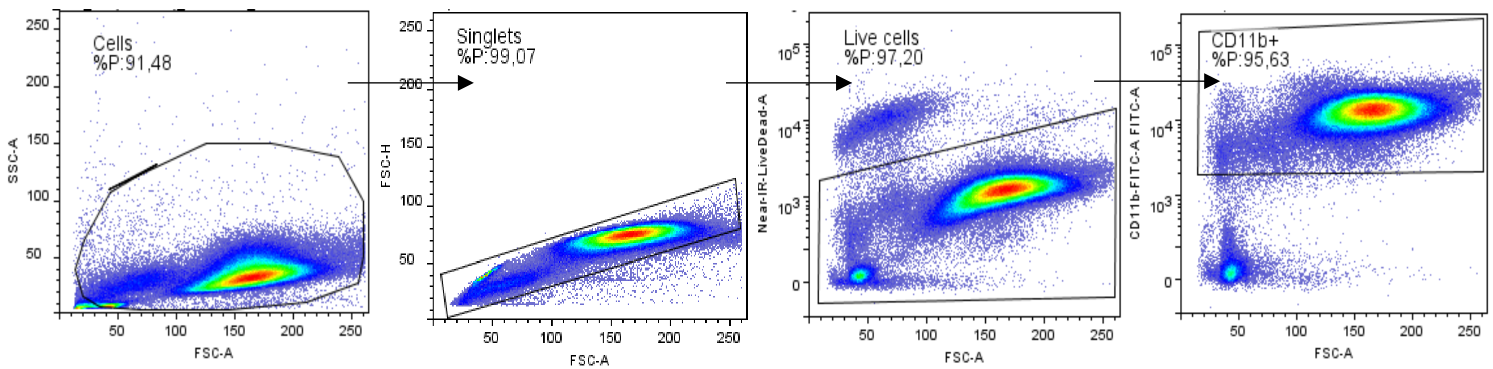

Figure S2. Gating strategy.
